# Pan-H7 influenza human antibody virus neutralization depends on avidity and steric hindrance

**DOI:** 10.1172/jci.insight.186182

**Published:** 2025-06-05

**Authors:** Iuliia M. Gilchuk, Jinhui Dong, Ryan P. Irving, Cameron D. Buchman, Erica Armstrong, Hannah L. Turner, Sheng Li, Andrew B. Ward, Robert H. Carnahan, James E. Crowe

**Affiliations:** 1 The Vanderbilt Center for Antibody Therapeutics, Vanderbilt Medical Center, Nashville, Tennessee, USA.; 2 Department of Integrative Structural and Computational Biology, The Scripps Research Institute, La Jolla, California, USA.; 3 Department of Medicine and Biochemical Sciences, School of Medicine, University of California, San Diego, California, USA.; 4 Department of Pediatrics and; 5 Department of Pathology, Microbiology and Immunology, Vanderbilt University Medical Center, Nashville, Tennessee, USA.

**Keywords:** Immunology, Infectious disease, Virology, Adaptive immunity, Influenza

## Abstract

H7N9 avian influenza virus is a zoonotic influenza virus of public health concern, with a 39% mortality rate in humans. H7N9-specific prevention or treatments for humans have not been approved. We previously isolated a human monoclonal antibody (mAb) designated H7-235 that broadly reacts to diverse H7 viruses and neutralizes H7N9 viruses in vitro. Here, we report the crystal structure of H7 HA1 bound to the fragment antigen-binding region (Fab) of recombinant H7-235 (rH7-235). The crystal structure revealed that rH7-235 recognizes residues near but outside of the receptor binding site (RBS). Nevertheless, the rH7-235 IgG potently inhibits hemagglutination mediated by H7N9 viruses due to avidity effect and Fc steric hindrance. This mAb prophylactically protects mice against weight loss and death caused by challenge with lethal H7N9 viruses in vivo. rH7-235 mAb neutralizing activity alone is sufficient for protection when used at a high dose in a prophylactic setting. This study provides insights into mechanisms of viral neutralization by protective, broadly reactive anti-H7 antibodies, informing the rational design of therapeutics and vaccines against H7N9 influenza virus.

## Abstract 2

This paper describes the structure and mechanism of action of a unique human monoclonal antibody that potently inhibits H7N9 avian influenza virus.

## Introduction

Avian influenza A virus (IAV) H7N9 strains have caused 5 epidemic waves (during the period between 2013 and 2017) and infected at least 1,567 humans, resulting in severe respiratory illness and death in approximately 40% of reported cases (1). In addition to H7N9 viruses, human infections with H7N2, H7N3, H7N4, and H7N7 viruses have been reported but cause isolated cases of mainly mild to moderate respiratory illness or conjunctivitis. H7N9 viruses predominantly infect birds because of the receptor specificity for sialic acid with α2-3 linkage that is typical in birds. Investigators have shown, however, that H7N9 viruses can acquire human–type α2-6 sialic acid–linked receptor specificity by mutating three additional amino acid (aa) residues within the receptor binding site (RBS) (2). The possibility of human-to-human transmission of H7N9 viruses presents a public health threat because these viruses are antigenically distinct from seasonal IAVs, and the human population generally lacks protective immunity against them. Despite differences in antigenicity, H7N9 virus elicits a human B cell response directed to immunodominant HA head domain, similar to the recognition patterns of seasonal IAVs (3). The RBS, antigenic sites A and B, and the trimer interface-II region were identified as the main regions recognized by human antibodies on the head domain after H7N9 infection (4, 5) or vaccination (6). Many site-A–targeting murine and human mAbs have been isolated and some exhibit potent neutralizing, HAI, and in vivo protective activities. Despite the relatively large number of published structures of antibody/H7 HA complexes available, however, a structure for a neutralizing mAb targeting the H7N9 antigenic site A has not been reported.

Here, we determined the crystal structure of the HA1 subunit of the surface HA protein of the avian H7N9 (SH13) influenza virus in complex with the Fab of antigenic site A-targeting mAb designated rH7-235 IgG.

## Results

**Figure 1:**
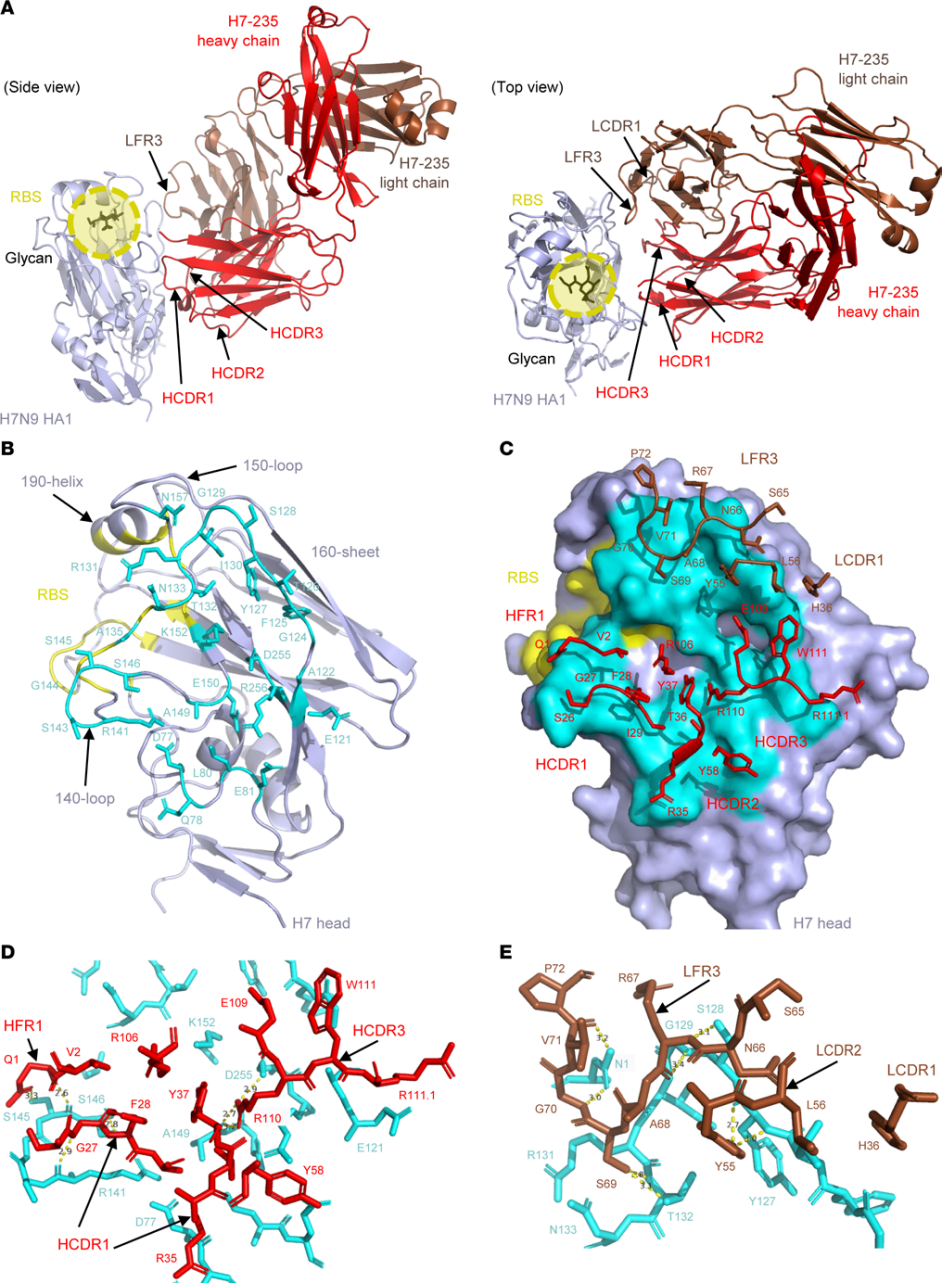
Crystal structure and detailed interactions between Fab H7-235 and H7N9 SH13 HA1. (**A**) Cartoon representation of the structure of the antigen-antibody complex. The HA1 domain is colored light blue, the receptor binding site (RBS) is indicated as a yellow circle, the H7-235 Fab heavy chain is red, and the light chain is brown. Individual CDRs of Fab H7-235 are labeled. Left or right panels show side or top views of the complex structure. See also Supplemental Figure 1, B and C and Supplemental Table 1. (**B**) Cartoon representation of H7-235 binding site. The HA1 domain is colored light blue, the RBS is yellow, and H7-235 Fab contact residues on HA1 are indicated with side chains and cyan color. See also Supplemental Figure 1A. (**C**) The H7-235 paratope. The footprint of H7-235 on the H7 HA surface is labeled in cyan color. The H7-235 Fab heavy chain is red, and the light chain is brown. H7-235 heavy or light chain contact residues are indicated with side chains in red or brown, respectively. The heavy chain and light chain interactions are shown in detail in **D** and **E**, with slightly reoriented views. Yellow dash lines represent hydrogen bonds in heavy or light chains.

MAb H7-235 was isolated from a B cell of an otherwise healthy individual following recovery from naturally acquired infection with a WT influenza A H7N9 virus (4). MAb H7-235 exhibited a pan-H7 subtype binding pattern, hemagglutination inhibiting activity (HAI), and neutralizing activity. To understand the molecular mechanisms of H7 HA neutralization mediated by H7-235, we performed negative stain electron microscopy (nsEM), hydrogen-deuterium exchange mass spectrometry (HDXMS) (Supplemental Figure 1A; supplemental material available online with this article; https://doi.org/10.1172/jci.insight.186182DS1), and determined the crystal structure of rH7-235 Fab in complex with a recombinant form of the HA1 subunit of A/Shanghai/2/2013 H7N9 HA at a 2.5-Å resolution (Supplemental Table 1 and Figure 1). The HA1 protein contained residues T12 to V311, with positions assigned in the H3 numbering scheme by an HA-subtype numbering conversion software program HA Subtype Numbering Conversion (Beta). The asymmetric unit (ASU) contained 2 copies of the monomeric HA1 protein bound to 1 rH7-235 Fab each. The 2 copies of the complex in the structure were superimposable with a root mean square deviation (RMSD) of 0.701 Å for the Cα atoms. Analysis of data obtained from the crystal structure of the H7-235 Fab/HA1complex revealed that rH7-235 recognizes residues outside of RBS but near the edge of the RBS on the side of the H7 head domain (Figure 1A). Close examination of the antigen-antibody interface determined using a 5 Å distance cutoff showed that the antibody binding site consists of 28 residues located on the membrane distal domain of HA between the HA protein 140 loop and 160 sheet (Figure 1B). The rH7-235 Fab interacts with HA1 using contacts in both heavy and light chains (Figure 1, C–E). The paratope on the Fab comprises 26 residues. The Fab heavy chain complementarity determining region 3 (HCDR3) residue R110 (ImMunoGeneTics information system numbering) contacts three HA1 residues: A149, E150 and D255 (Figure 1D). The HCDR1 and heavy chain framework region 1 (HFR1) interact with the HA 140 loop: Fab residue G27 with HA1 residues R141 and S146 and Fab residue Q1 with HA1 residue S145. Antibody light chain framework region 3 (LFR3) residues Y55, R67, S69, G70, and V71 are encoded by the IGKV2-28*01 germline gene segment and contact HA1 residues Y127, S128, G129, T132, or N157, respectively (Figure 1E). The complex interface buried an average of 929.2 Å^2^ surface area with the interacting residues of the heavy chain of H7-235 occupying an average of 609.4 Å^2^ surface area (66% of total) compared with the light chain of rH7-235, which contributes an average of 319.8 Å^2^ surface area (34% of total). The results of nsEM and HDXMS are in accordance with the crystal structure. The 2D and 3D nsEM analyses also provide more insights into stoichiometry of rH7-235 Fab interaction with trimeric soluble HA, since HA:Fab complexes with three Fabs are bound to one HA trimer detected in solution (Supplemental Figure 1, B and C).

**Figure 2:**
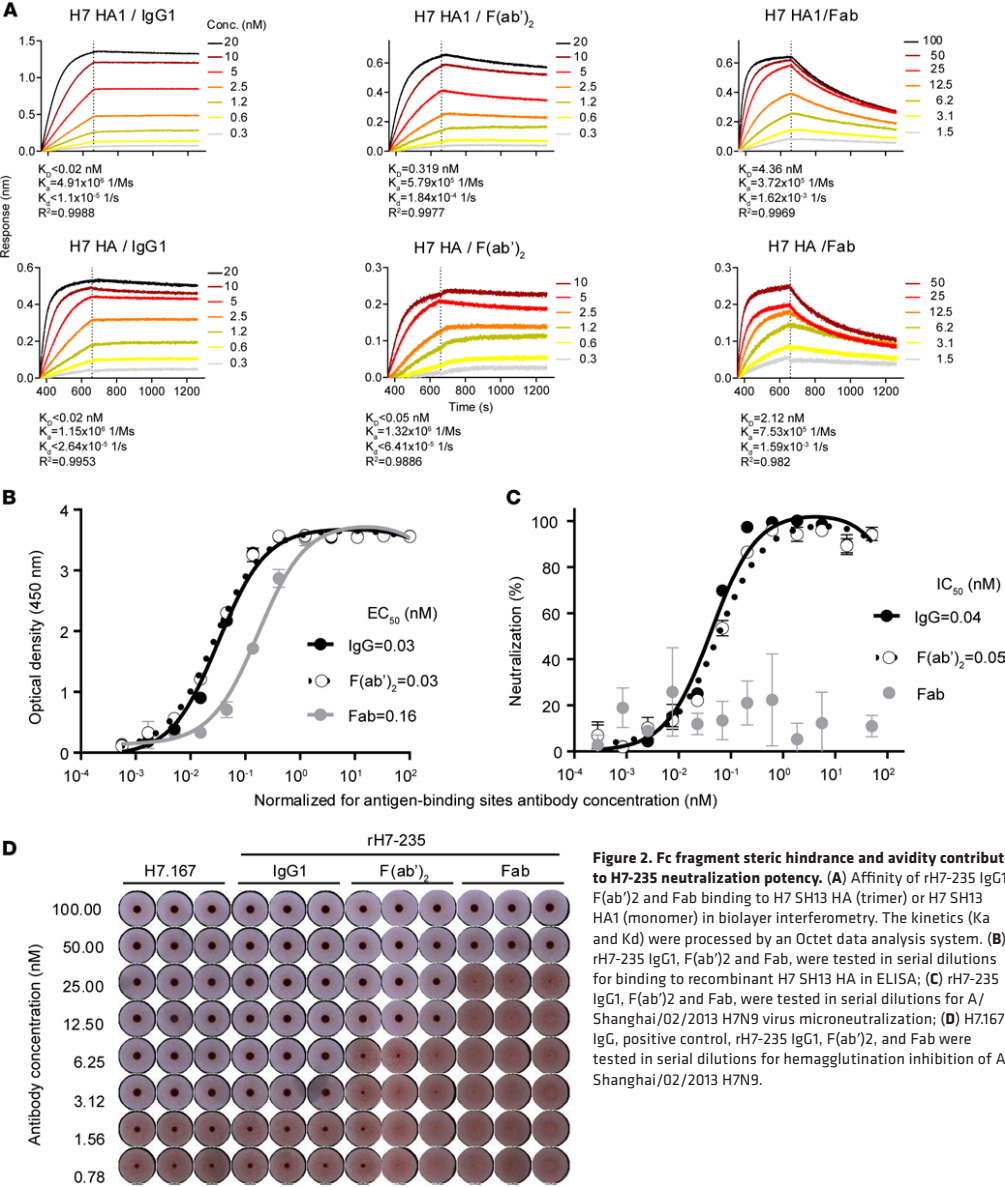
Fc fragment steric hindrance and avidity contribute to H7-235 neutralization potency. (**A**) Affinity of rH7-235 IgG1, F(ab′)2 and Fab binding to H7 SH13 HA (trimer) or H7 SH13 HA1 (monomer) in biolayer interferometry. The kinetics (Ka and Kd) were processed by an Octet data analysis system. (**B**) rH7-235 IgG1, F(ab′)2 and Fab, were tested in serial dilutions for binding to recombinant H7 SH13 HA in ELISA; (**C**) rH7-235 IgG1, F(ab′)2 and Fab, were tested in serial dilutions for A/Shanghai/02/2013 H7N9 virus microneutralization; (**D**) H7.167 IgG, positive control, rH7-235 IgG1, F(ab′)2, and Fab were tested in serial dilutions for hemagglutination inhibition of A/Shanghai/02/2013 H7N9.

The crystal structure of the complex formed by the rH7-235 Fab with H7 HA1 shows that the antibody does not contact the RBS and it does not clash with the substrate glycan within RBS (Figure 1). Nevertheless, the rH7-235 IgG exhibits very potent HAI activity for H7N9 virus (with a maximal inhibitory value [IC_100_] of approximately 120 ng/mL) (Figure 2D and reference (4)), which is comparable to the potency of the mAb H7.167 that directly blocks glycan binding (7). The importance of the Fc region for the neutralizing activity of rH7-235 is suggested by the fact that IgG, F(ab)_2_, and Fab forms of rH7-235 neutralize virus at increasingly higher molar concentrations of 3.1, 12.5, or 50 nM, respectively. The reduction of neutralization activity for the Fab form of H7-235 can be partially explained by a loss of avidity. It should be noted, however that the F(ab)_2_ form of H7-235 exhibited reduced neutralization activity despite F(ab)_2_ binding to hemagglutinin in ELISA (Figure 2B), and biolayer interferometry (BLI) (Figure 2A) assays were comparable to that of IgG. So, the HAI activity of full-length mAb H7-235 IgG is facilitated by both favorable avidity of interaction in the bivalent molecule and Fc steric hindrance effects achieved only by full-length IgG. In contrast to HAI, the Fab did not neutralize virus at any tested concentration in microneutralization, and F(ab)_2_ and IgG were similarly potent (Figure 2C). This result suggests that avidity plays a major role in virus neutralization on Madin-Darby Canine Kidney (MDCK) cell line monolayers.

**Figure 3:**
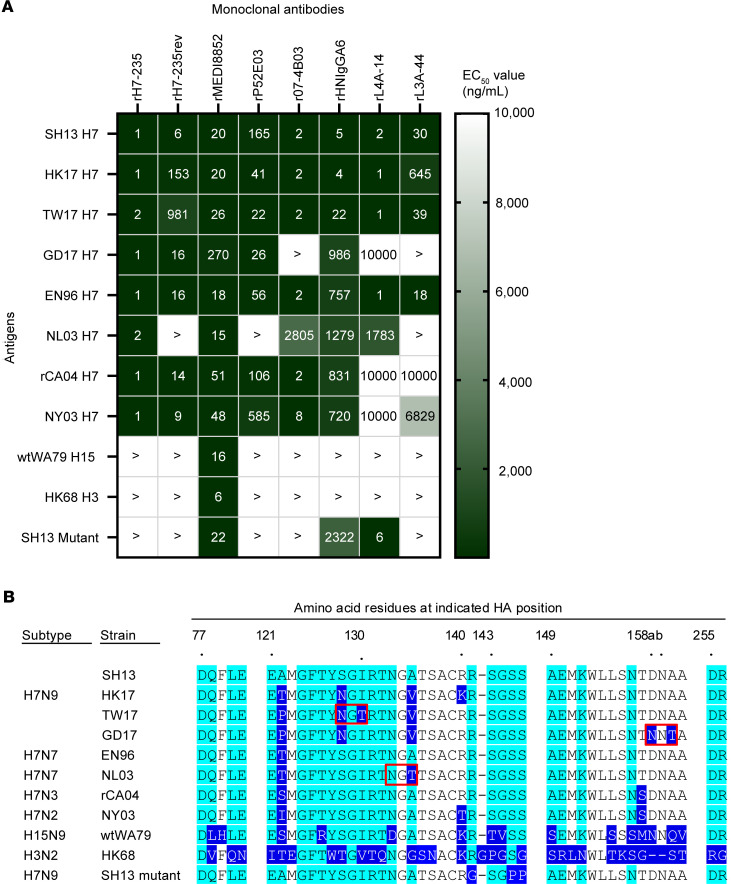
H7-235 is a broadly reactive mAb and sensitive to aa substitutions within the antigenic Site A. (**A**) Heatmap for cross-reactivity of mAbs rH7-235 IgG1, rH7-235rev IgG1, rMEDI8852 IgG1, rP52E03 IgG1, r07-4B03 IgG1, rHNIgGA6 IgG1, rL4A-14 IgG1, and rL3A-44 IgG1 with recombinant soluble H7 HA proteins from A/Shanghai/02/2013 (SH13 H7), A/Hong Kong/1/2017 (HK17 H7), A/Taiwan/01/2017 (TW17 H7), A/Guangdong/8H324/2017 (GD17 H7), A/England/268/1996 (EN96 H7), A/Netherlands/219/2003 (NL03 H7), A/Canada/rv504/2004 (rCA04 H7), A/New York/107/2003 (NY03 H7), A/shearwater/Western Australia/2576/1979 (wtWA79 H15), A/Hong Kong/1/1968 (HK68 H3), and triple mutant R141G/S145P/S146P on SH13 (SH13 Mutant). Representative EC_50_ values (ng/mL) from one of two independent experiments are plotted. See also Supplemental Figures 2 and 3. > indicates more than 10,000 ng/mL. (**B**) Alignment aa sequences of HA from multiple H7Nx, H15N9, and H3N2 influenza viruses within H7-235 epitope. Contact residues are highlighted with cyan color, aa substitutions are highlighted with blue color, and glycosylation sites acquired by H7Nx IAVs are indicated with red boxes.

Previously, we reported that mAb H7-235 competes for binding to HA with antigenic site A antibodies in competition-binding assays (4). To check here if binding with the HA 140-loop region is critical for recognition by mAb rH7-235, we tested mAb rH7-235 binding to a variant HA molecule with triple R141G/S145P/S146P mutations. The R141G was selected as an escape mutation for many murine and human site A-specific mAbs, including mAbs 1A8 (8), 2C4, 4A2, 5A6 (9), and 07-4D05 (6). The S145P or S146P HA mutations were observed for escape mutants selected with the clonally related human mAbs 07-4B03 or 07-4E02, respectively (6). rH7-235 is seen to contact these three residues in the 140-loop in the solved crystal structure of rH7-235 in complex with H7 HA1. For comparison, we analyzed reactivity to known triple-mutant head-domain targeting neutralizing human mAbs as introduced above, r07-4B03 and rP52E03 (10), rHNIgGA6 (11), rL4A-14, and rL3A-44 (5). The R141G/S145P/S146P mutations abrogated binding of mAb rH7-235. Although, the same mutations resulted in loss of binding for all head-targeted mAbs tested except rL4A-14 (Figure 3, A and B). This general loss of binding pattern can be explained by the fact that all these mAbs interact with the 140-loop to some extent. Thus, rH7-235, rP52E03, and r07-4B03 interact with all three mutated residues, and rHNIgGA6 and rL3A-44 interact with S145. MAb rL4A-14 interacts only with the S143 residue in the 140-loop, so this mAb retains binding to the HA triple mutant R141G/S145P/S146P. This result suggests the 140 loop plays an important role in rH7-235 antibody interaction with H7 HA.

The rH7-235 IgG binds broadly to many H7 HA molecules representing antigenically diverse naturally occurring WT H7N9 field strains (Figure 3A). The breadth of reactivity of rH7-235 exceeds that of other anti-H7 head-domain–reactive potently neutralizing mAbs, like rHNIgGA6, rP52E03, r07-4B03, rL3A-44, and rL4A-14. To understand the basis for broad recognition of these strains by rH7-235 mAb, we aligned HA aa sequences from representative H7 strains (Figure 3B). Among the 28 residues recognized by rH7-235 (highlighted in cyan on Figure 3B) are peptide regions containing aa121–135 and aa141–146. The aa121–135 region contains four substitutions, A122T/P/S/I, S128N, I130T, and A135V/T, among representative IAV strains that are well tolerated by rH7-235 mAb (Figure 3B). Binding of rH7-235 mAb also was not affected by glycosylation of the asparagine residue at positions N133 in the HA of A/Netherlands/219/2003 (NL03 H7N7), N128 of A/Taiwan/1/2017 H7N9 (TW17 H7N9), or N158a of A/Guangdong/8H324/2017 H7N9 (GD17 H7N9). However, the majority of RBS-targeted potent mAbs, including mAbs rP52E03, r07-4B03, rHNIgGA6, rL4A-14, and rL3A-44, are sensitive to glycosylation of these asparagine residues, especially in position N133 (NL03 H7N7), as shown in Figure 3A. Glycosylation in NL03 H7N7 (N133) reportedly prevents binding of many neutralizing mAbs targeting site A (12). rH7-235 reactivity does not extend to H15 or H3 subtype HAs, likely because these proteins have many substitutions within the rH7-235 epitope, even though they belong to the same phylogenetic Group 2 as H7. These data explain the pan-H7 cross-reactive recognition pattern of rH7-235 and its lack of heterosubtypic binding to H15 or H3 HAs.

We also measured the binding properties of a germline revertant form of rH7-235 IgG. The germline revertant sequence is shown in Supplemental Figure 2. rH7-235 and its germline revertant bind comparably to SH13 H7 HA. However, the revertant is more sensitive to introduction of single glycosylation sites (TW17 H7N9 or NL03 H7N7) and aa substitutions (A/Hong Kong/125/2017 H7N9 (HK17 H7N9)) within and/or near the epitope. Thus, the antigenic site A contains important sites of vulnerability for neutralization recognized by the human immune system.

**Figure 4:**
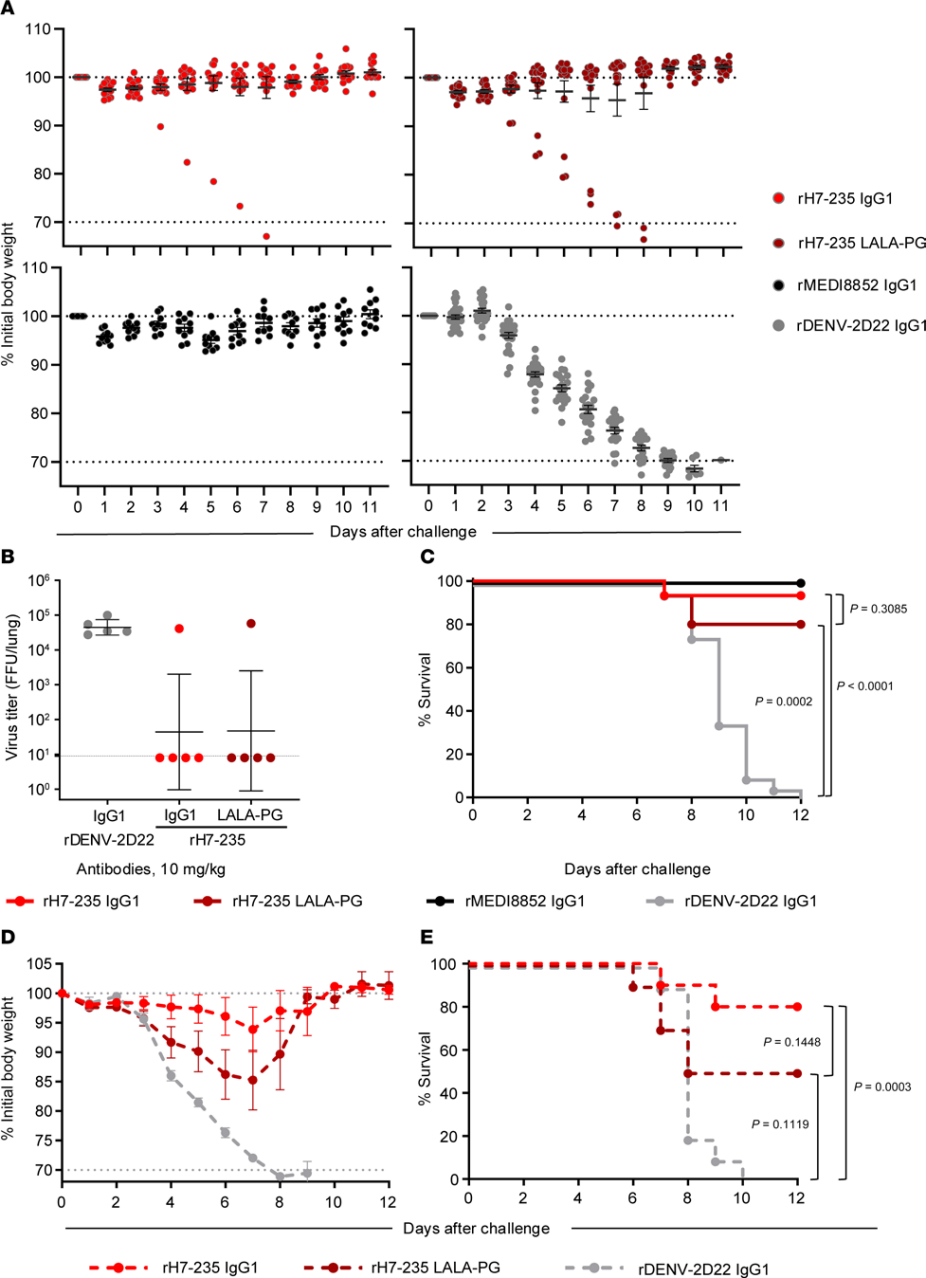
rH7-235 mAb is protective in vivo and Fc-effector function independent at higher mAb dose. (**A**–**C**) Female 18-20 g BALB/c mice were inoculated by the intraperitoneal route with a 10 mg/kg (**A**-**C**) or 2 mg/kg (**D**-**E**) dose of mAbs: (**A**-**C**) rH7-235 IgG1 (*n* = 20), rH7-235 LALA-PG (*n* = 20) or a control antibody (DENV 2D22 [negative control] (*n* = 25) or rMEDI8852 [positive control] (*n* = 10); (**D**-**E**) rH7-235 IgG1 (*n* = 10), rH7-235 LALA-PG (*n* = 10) or DENV 2D22 *(n* = 10). At 24 hours after the mAb treatment, anesthetized mice were inoculated by the intranasal route with 10^4^ focus forming units of H7N9 SH13 (IDCDC-RG32A) virus in 50 µL of sterile PBS. Mice were weighed and monitored daily for body weight change (**A**, **D**) and survival (**C**, **E**) for 12 days. Those losing over 30% of initial body weight were euthanized humanely as per IACUC requirements. To determine viral replication in lungs of infected mice, 5 animals from H7-235 IgG, LALA-PG and negative control groups were euthanized on day 3 post-infection (**B**). Data shown are mean with SEM. Comparison of survival curves performed using Kaplan-Meier Method with Log-rank (Mantel-Cox) test using GraphPad Prism 10.3.1. See also Supplemental Figure S4.

We next sought to determine whether the in vitro neutralizing activities of mAb rH7-235 correlate with prophylactic protection efficacy in vivo and to test whether Fc-mediated antibody effector functions contribute to protection. We tested treatment with rH7-235 IgG1 or a variant Fc region rH7-235 IgG with LALA-PG mutations that reduced ability to bind to Fc receptors in a lethal H7N9 mouse model of infection (Figure 4, A–C). rH7-235 LALA-PG had comparable in vitro binding and HAI activity to rH7-235 IgG1, as shown in Supplemental Figure 4. We injected 8-week-old female BALB/c mice (*n* = 10 to 20 per group) by the intraperitoneal route with 200 μg (10 mg/kg) of mAb 24 hours before intranasal challenge with 1 × 10^4^ focus forming units of H7N9 SH13 (IDCDC-RG32A) virus in 50 μL of PBS. Mice were monitored daily for weight loss and lethality (with 30% of weight loss considered as an endpoint for euthanasia, per IACUC requirements). All (*n* = 20) mice treated with an isotype-matched control human mAb (rDENV-2D22 IgG1, directed to dengue virus envelope protein (13)) lost weight and were euthanized up to day 12. In contrast, only 1 of 15 animals treated with mAb rH7-235 IgG1 and 3 of 15 animals treated with rH7-235 LALA-PG mAb showed weight loss and were euthanized. So, the rH7-235 LALA-PG antibody protection of mice against lethal challenge with H7N9 at a dose of 10 mg/kg is comparable to that mediated by H7-235 IgG1, suggesting that the neutralization potency of the mAb is the most critical feature contributing to protection. The viral titers in lung tissue harvested from mice treated with H7-235 IgG1, LALA-PG, and DENV-2D22 IgG1 on day 4 after viral infection were consistent with the data for weight loss and protection from lethality, with one outlier in both H7-235 IgG1 and LALA-PG-treated groups, which had a lung viral titer comparable with that in the negative control treated group. When the H7-235 IgG1 and LALA-PG mutant were delivered at a lower (2 mg/kg) dose 1 day before virus challenge, only rH7-235 IgG1-treated animal group differed significantly from the isotype-matched control IgG-treated group. At the lower (2 mg/kg) dose of rH7-235 IgG1, Fc-effector function may play a minor role in the animal protection, though this effect was not statistically significant in the conditions tested (Figure 4, D and E). These results suggest that the neutralizing activity of rH7-235 mAb alone is sufficient for protection when high-dose treatment is used in the prophylaxis setting.

## Discussion

**Figure 5:**
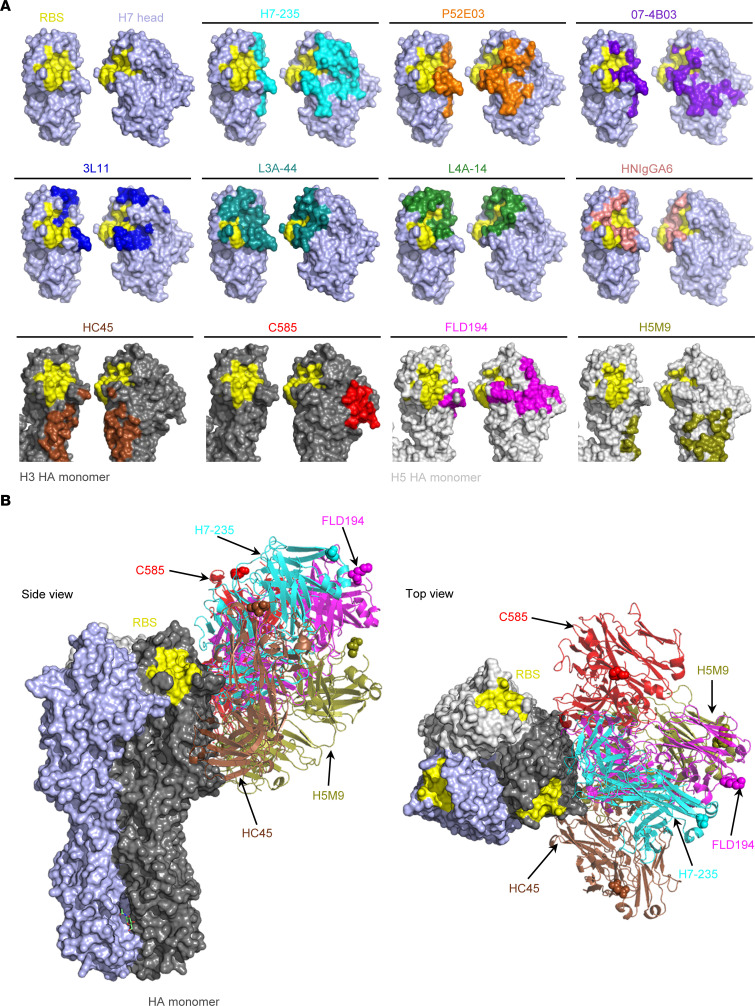
Comparison of rH7-235 and other antibodies targeting H7 HA head and HA head (non RBS) showing differences in footprint and approach. (**A**) MAb footprints indicated in colors: H7 235 (cyan), P52E03 (orange), 07 4B03 (purple), L4A 14 (green), L3A 44 (teal), HNIgGA6 (salmon), FLD194 (magenta), H5M9 (olive), C585 (red), and HC45 (brown), peptide interacting with 3L11 (blue) on surface representation of H7 head (light blue), H5 HA monomer (light gray), and H3 HA monomer (gray). Yellow color indicates RBS. (**B**) Superimposition of the H7-235 Fab H7 HA1 complex structure and the C585 H3 HA, HC45 Fab H3 HA, H5M9 Fab H5 HA, and FLD194 Fab H5 HA complex structures onto H3N2 HA trimer. Structures of the H5 HA and the H7N9 HA1 domain in the complexes were omitted for clarity. The H3 HA protomer, onto which the complexes were superimposed, is shown in gray. Yellow color indicates RBS. Fabs have shown in cartoon representation with spheres indicating last residue of Fab C_H_1, pointing to location of Fc hinge in IgG.

Antibodies to influenza A H7 HA protein serve as the principal correlate of protection against disease and infection, but the molecular basis of recognition of H7 HA by human antibodies is poorly understood. The H7 HA head domain antigenic site A is a dominant target of neutralizing antibodies, and many anti-H7 HA mAbs are sensitive to mutations of 5 key residues (R141/S143/G144/S145/S146) that are highly conserved in H7 proteins. Some of these antibodies show cross reactivity to divergent H7 viruses, possess HAI and neutralizing activities, and protect in vivo. Many previously reported murine mAbs such as 1B2 (8), 5A6, 2C4, 4A2, and 1A8 (9), and human mAbs 3L11 (14), H7.169 (7), 07-4D05, 07-4E02, 07-4B03 (6), and P52E03 (10) select escape mutant viruses with amino acid changes at 5 key residues of antigenic site A. To date, there is limited insight on how this type of antibody interacts with H7 HA. So far, only computational docking-based predictions provided models of P52E03/H7 and 07-4B03/H7 complex structures (10), and some mass spectrometry studies identified a linear peptide immunoprecipitated by mAb 3L11 (14). rH7-235 mAb binding to H7 HA is abrogated by mutations R141G/S145P/S146P. The abrogation of mAbs rHNIgGA6 and rL3A-44 binding to H7 HA by a site A triple mutant highlight difficulties with mapping antibody epitopes solely based on tolerance for single aa substitutions. The cocrystal structures of complexes HNIgGA6/H7 (11) and L3A-44/H7 (5) revealed these mAbs mostly interact with the 130 loop, 190 helix, and 220 loop surrounding RBS, and these are not antigenic site A–targeting mAbs. On the other hand, the putative epitopes for P52E03 and 07-4B03 largely overlap the rH7-235 mAb epitope (Figure 5A) and share 15 or 10 residues on H7 HA with the rH7-235 footprint, respectively. The interactions of these antibodies differ from rH7-235 mAb in that both P52E03 and 07-4B03 directly interact with the RBS. As a result, the binding of these mAbs is sensitive to substitutions near the RBS, such as the N133 glycosylation that was acquired naturally in some H7 IAV strains, like NL03 H7N7. In contrast, the receptor-blocking (HAI) activity of H7-235 works by steric hindrance conferred by the antibody Fc fragment region. Several other human mAbs, which neutralize virus hemagglutination activity and protect animals without directly contacting the RBS, have been previously reported, including the anti-H7 mAb 3L11 (14), the anti-H3 mAbs HC45 (15) and C585 (16), and the anti-H5 mAb FLD194 (17) and H5M9 (18). When comparing the footprint of rH7-235 with those of the latter mAbs, we note that mAbs 3L11 and FLD194 share the most similarity in terms of the contact region on the corresponding HA (Figure 5A). There are few details on the 3L11 epitope except that the mAb binds to an aa141–167 peptide of HA, and we note that rH7-235 contacts 7 residues within this peptide. The epitopes recognized by rH7-235 and FLD194 differ substantially due to amino acid sequence differences between H7 and H5 HA proteins. Detailed information on the mechanism of HAI is not available for 3L11, but mAb FLD194 reportedly neutralizes virus through steric hindrance cause by its Fc region, similar to rH7-235. Also, as in the case with rH7-235, the Fab form of FLD194 exhibited reduced HAI activity and substantial loss of neutralization activity compared with the IgG form. FLD194 IgG crosslinks neighboring HAs on HA rosettes, as shown by electron microscopy, and the authors suggested that an IgG layer positioned above the HA restricts access of the virus to cell-surface sialic acid receptors, sterically preventing virus attachment (17).

Despite the wide range of epitopes recognized by mAbs that mediate HAI activity due to steric hindrance, as first noted in Qiu et al. (16), the common feature of all such mAbs is that their Fc region is oriented upward toward the host cell receptor (Figure 5B). Of course, antibody Fc-mediated protection also can be achieved in some cases by engaging Fc receptors on immune cells. For instance, mAb 3L11 demonstrated antibody-dependent cell-mediated cytotoxicity in vitro. This activity has not been tested in vivo for any of the non-RBS HAI+ antibodies discussed above. Here, we tested mAb rH7-235 for Fc-mediated effector function contribution in protection in vivo using the Fc variant LALA-PG IgG and showed that at high concentrations rH7-235 IgG1 provides Fc-effector–independent protection in vivo.

Further research is needed to explore the reason for the difference between rH7-235 Fab neutralizing potential in HAI and microneutralization tests, and to exclude the possibility that rH7-235 IgG possesses additional effects related to attachment, fusion and/or egress. First, the host cell receptors on turkey red blood cells (TRBCs) or MDCK used in these two assays may differ by receptor composition, length, and avidity of interaction with H7N9 virus, and, consequently, these RBCs may differ in sensitivity to steric hindrance driven by Fab or IgG. A difference between results in HAI and microneutralization assays due to difference in viral receptor-binding avidity of H7N9 have been reported previously (19). Second, the microneutralization assay tests mAb effects on several steps in the virus life cycle, including attachment and fusion; therefore, we can conclude the rH7-235 Fab does not contribute to blocking either of those steps. The HAI assay is a standard method for attachment inhibition characterization of antibodies with live influenza viruses. HAI also measures blocking attachment avian receptors when using TRBCs, and avian receptors are the natural receptors for H7N9 viruses. In summary, we determined the structural basis of molecular recognition for a broadly reactive (“pan-H7”) human antibody that mediates receptor blocking (in vitro HAI activity) without contacting the RBS directly by solving a cocrystal structure of rH-235 Fab/H7 HA1. And we showed the mechanisms of viral inhibition mediated in vivo by rH7-235 are complex and involve both indirect receptor blocking and Fc effector functions.

## Methods

### Sex as a biological variable.

mAb characterized in this study were derived exclusively from one healthy male donor. Sex was not considered as a biological variable during donor recruitment, and it is unknown whether these findings reflect the heterogeneity of the influenza-specific humoral response found in females. Furthermore, this study exclusively examined protection from lethality in female mice. Sex was not considered as a biological variable in mice, and it is unknown whether these findings are relevant for male mice.

### Cell lines and viruses.

The reverse-genetics–derived virus with the H7N9 HA and NA genes on a PR8 backbone, designated A/Shanghai/2/2013 (H7N9)-PR8-IDCDC-RG32A (Influenza Reagent Resource, FR-1389), was propagated in embryonated chicken eggs and manipulated under BSL-2 conditions with BSL-3 practices. Virus titration and neutralization assays were performed on MDCK (ATCC, CCL-34) epithelial cells. Cells were determined to be mycoplasma free at laboratory passage 2, and monthly when in use.

### Recombinant antigens expression and purification.

A cDNA encoding A/Shanghai/02/2013 H7N9 (SH13) hemagglutinin HA1 (residue 1–320, cognate signal peptide included) and a 6-his tag was synthesized (Genscript) and cloned into pcDNA3.1(+) (Thermo Fisher; V79020). A cDNA encoding the HA genes from the IAVs A/Shanghai/02/2013 H7N9, A/Hong Kong/125/2017 H7N9, A/Taiwan/01/2017 H7N9, A/Guangdong/8H324/2017 H7N9, A/England/268/1996 H7N7 and A/Netherlands/219/2003 H7N7, A/Canada/rv504/2004 H7N3 and A/New York/107/2003 H7N2, A/shearwater/Western Australia/2576/1979 H15N9, A/Hong Kong/1/1968 H3N2, and triple mutant R141G/S145P/S146P on SH13 backbone were optimized for mammalian expression. The cDNAs were synthesized (GenScript) as soluble trimeric constructs by replacing the transmembrane and cytoplasmic domain sequences with cDNAs encoding the GCN4 trimerization domain and a His6-tag at the C-terminus. Synthesized genes were subcloned into the pcDNA3.1(+) mammalian expression vector (Thermo Fisher Scientific). HA proteins were expressed by transient transfection of FreeStyle 293-F cells (Thermo Fisher Scientific, R79007) with polyethyleneimine transfection reagent and was grown in expression medium (FreeStyle 293 Expression Medium; Thermo Fisher Scientific). The supernatants were harvested after 7 days, filter-sterilized with a 0.4 μm filter, and purified by nickel affinity chromatography with HisTrap Excel columns (GE Healthcare Life Sciences).

### Recombinant Fab and antibody cloning, expression, and purification.

For the expression of recombinant Fab and IgG1, nucleotide sequences of antibodies H7-235, MEDI8852 (20), P52E03, 07-4B03, HNIgGA6, L4A-14, and L3A-44 heavy- and light-chain variable genes were codon optimized for mammalian expression and synthesized at Twist Biosciences. The resulting gene fragments were directly cloned at Twist Biosciences into the pTwist CMV BetaGlobin WPRE NEO mammalian expression vector (Twist Biosciences) with C_H_1/C_L_ for Fab and C_H_1/C_L_ with Fc fragment for IgG1. The expression vectors were transfected to Expi293F (ThermoFisher Scientific, A14528) cells transiently, and supernatants were harvested after culturing for 6 to 7 days. Recombinant Fab H7-235 was purified with Anti-CH1 CaptureSelect column (GE Healthcare Life Sciences). Recombinant IgG1 antibodies were purified with MabSelect Sure column (GE Healthcare Life Sciences).

The F(ab)_2_ have been generated by enzymatic cleavage using IdeS Protease (Promega, V7511) according to vendor protocol.

### Hemagglutination inhibition.

The HAI assay was performed with A/Shanghai/2/2013 (H7N9)-PR8-IDCDC-RG32A. For HAI, 25 μL of 4 HA units of virus were incubated for 1 hour at room temperature (RT) with 25 μL 2-fold serial dilutions of antibodies starting at 10 μg/mL and equimolar amounts of F(ab)_2_ or Fab in PBS. The 50 μL of antibody-virus mixture was incubated for 45 minutes at 4°C with 50 μL of turkey red blood cells (Rockland Immunochemicals) diluted in PBS. The IC_100_ value was defined as the lowest antibody concentration that inhibited hemagglutination of red blood cells.

### Microneutralization.

The microneutralization assay was performed on MDCK cell line monolayers, seeded in 96-well plates one day before experiment, using A/Shanghai/2/2013 (H7N9)-PR8-IDCDC-RG32A under BSL-2 conditions with BSL-3 practices. For assay, pretitered virus was incubated for 1 hour at 37°C with 3-fold serial dilutions of antibodies starting at 100 nM of IgG1, F(ab)_2_, or Fab in 1 × DMEM/2% BSA media. The MDCK cells washed 3 times with PBS and 100 μL of antibody-virus mixture was applied to them. Virus was allowed to attach to MDCK cells for 1 hour at 37°C. After virus was removed from cells and cells were overlaid with 1.2% Avicel (FMC) prepared using NaHCO_3_-buffered serum-free 2 × DMEM and supplemented with TPCK-treated trypsin (1 μg/mL). Endpoint virus titers were determined by visualizing virus foci 24 hours after infection by removing overlay, inactivating virus, and permeabilizing cells with 80% methanol in PBS for 20 minutes at RT, and blocked by PBS/ 0.05% Tween-20 PBS containing 5% milk (BioRad, #1706404) for 1 hour at RT, stained with anti-Influenza A Nucleoprotein mouse (A1+A3) antibodies (BEI Resources, NR-4282, 1/6,000 dilution) for 1 hour at RT, horseradish peroxidase-conjugated anti-mouse donkey’s antibodies (Novex, A16014, 1/500 dilution) for 1 hour at RT and TrueBlue HRP substrate (VWR, 95059-468). Plates were incubated with substrate for 10 minutes at RT. After, 1N HCl (Thermo Fisher Scientific, SA48-1) was added and the optical density values were measured at 450 nm wavelength on a BioTek plate reader. The plates were washed 3 times between each step with PBS containing 0.05% Tween-20. Each dilution was performed in triplicate, and the EC_50_ values were calculated in Prism software (GraphPad) using nonlinear regression analysis.

### ELISA.

To determine EC_50_ concentrations for binding, we performed ELISAs using 384-well plates that were coated overnight at 1 mg/mL with the recombinant HA protein of interest. The plates then were blocked with 50 μL of 0.1% Tween-20 in D-PBS (ELISA buffer) for 30 minutes at RT. The plates were washed and 3-fold dilutions of the mAbs in ELISA buffer at a starting concentration of 10 mg/mL or equimolar amounts of F(ab)_2_ or Fab were added to the wells and incubated for an hour. The plates were washed and 25 μL of ELISA buffer containing a 1:4,000 dilution of anti-human IgG horse radish peroxidase conjugate (Southern Biotech, 2040-05) was added. After a final wash, 1-Step ULTRA TMB-ELISA (Thermo Fisher Scientific, 34029) was added to the plates and incubated for 10 minutes at RT. After, 1N HCl (Thermo Fisher Scientific, SA48-1) was added, and the optical density values were measured at 450 nm wavelength on a BioTek plate reader. The plates were washed 3 times between each step with PBS containing 0.05% Tween-20. Each dilution was performed in triplicate, and the EC_50_ values were calculated in Prism software (GraphPad) using nonlinear regression analysis. Each experiment was conducted twice independently.

### Biolayer interferometry kinetics.

*K_D_* was determined by bio-layer interferometry (BLI) using an Octet Red instrument (FortéBio). SH13 H7 monomeric head and H7 trimeric HA ectodomain were biotinylated using EZ-Link NHS-PEG4-Biotin (Thermo Fisher Scientific, A39259) at an antigen:biotin ratio of 1:1 or 1:20, respectively. SH13 H7 head domain at 1.63 μg/mL or H7 HA at 1.5 μg/mL in 1 Kinetics buffer (FortéBio, 18-5032) was loaded onto Streptavidin (SA) biosensors (Sartorius, 18-5019) and incubated with varying concentrations of IgG1, F(ab)_2_ or Fab antibodies. All binding data were collected at 30 °C. The experiments comprised 4 steps: (a) antigen loading onto the biosensor; (b) baseline acquisition for 60 s; (c) association of antibodies for the measurement of *K_a_*; and (d) dissociation of antibodies for the measurement of *K_d_*. Baseline and dissociation steps were carried out in Kinetics buffer only. The ratio of *K_a_* to *K_d_* determined the value of *K_D_* reported here.

### In vivo protection study.

Mice were housed in individually ventilated cage racks with negative pressure ventilation and air filtering (Allentown Inc.). To assess protective efficacy of the mAb, female 18–20 g BALB/c mice (Charles River Laboratories) were inoculated by the intraperitoneal (i.p.) route with a 200-μg dose of H7-235 or a control antibody. Human anti-dengue virus mAb DENV 2D22 served as a mock control treatment, while a recombinant form of human mAb rMEDI8852 directed to the HA stem region served as a positive control. In ABSL-2+ facilities, ketamine-xylazine anesthetized mice were inoculated by the intranasal (i.n.) route at 24 hours after the mAb treatment with 1 × 10^4^ focus forming units (approximately 4 × LD80) of H7N9 A/Shanghai/2/2013 IDCDC-RG32A in 50 μL of sterile PBS. Mice were weighed and monitored daily for body weight change and signs of disease for 14 days, and those losing over 30% of initial body weight were humanely euthanized per IACUC requirements.

For lung titers, mice from rH7-235 IgG1, rH7-235 LALA-PG, and rDENV-2D22 IgG1 groups (*n* = 5) were sacrificed at 4 days after inoculation and lungs were removed aseptically, snap frozen on dry ice, and stored at −80°C until titration. Lungs were homogenized in 1 mL 1 × DMEM/2% BSA medium using a BeadBlaster 24 homogenizer (Benchmark). The homogenates were centrifuged (5 minutes, 10,000*g* at 4°C) to remove cellular debris and then were used for virus titration. A 250 μL volume of 10-fold dilutions of homogenized lungs in 1 × DMEM/2% BSA medium were used for inoculating confluent monolayers of MDCK cells. Virus was allowed to attach to MDCK cells for 1 hour at 37°C. Next, virus was removed from cells and cells were overlaid with 1.2% Avicel (FMC) prepared using NaHCO_3_-buffered serum-free 2× DMEM and supplemented with TPCK-treated trypsin (1 μg/mL). Endpoint virus titers were determined by visualizing virus foci 2 days after infection by staining with anti-Influenza A Nucleoprotein mouse antibody (A1+A3) (BEI Resources, NR-4282, 1/6,000 dilution), horseradish peroxidase-conjugated anti-mouse donkey’s antibodies (Novex, A16014, 1/500 dilution) and ELISpot substrate, TMB for HRP (MABTECH, 3651-10).

### Crystallization, data collection, and structure determination.

Purified H7N9 HA1 and H7-235 Fab were mixed in a molar ratio of 1:1.5. The mixture was incubated at room temperature for approximately 10 minutes, and then the antigen-antibody complex was purified from the mixture using a HiLoad 16/600 Superdex 200 size-exclusion column (GE Healthcare Life Sciences). The purified complex of H7N9 HA1 and H7-235 Fab was concentrated to approximately 10 mg/mL in a buffer of 20 mM Tris-HCl pH 7.5, 50 mM NaCl. Extensive crystallization screening was performed with the concentrated sample using a mosquito XTAL3 crystallization robot (TTP Labtech), and subsequent optimization was carried out with a dragonfly crystal screen optimizer (TTP Labtech). The H7-235/H7-HA1 complex was crystallized in 2 M NaCl, 8%–10% PEG 6000 and was cryoprotected using well solution:glycerol in a 20:7 ratio. Diffraction data were collected at the Advanced Photon Source LS-CAT beamline 21-ID-F. The data were processed and integrated with XDS data processing software package (21) and scaled with SCALA (22). The crystal structure of the complex was solved by molecular replacement with the software Phaser (23) using the crystal structure of H7N9 Shanghai head domain (PDB ID: 4N5J) and the crystal structure of human anti-Marburg virus Fab MR78 (PDB ID: 5JRP) as the searching models. Iterative refinement and manual rebuild of the crystal structure were done with Phenix and coot (24, 25). The data collection and refinement statistics are shown in Supplemental Table 1. All structure figures were made using the molecular graphics software PyMOL (Shrodinger).

### Negative stain electron microscopy.

H7-235 Fabs were incubated with uncleaved H7 SH13 HA trimer for 20 seconds at 5 times molar excess of Fab. The complex was added to carbon-coated 400 mesh cooper grids and stained with 2% uranyl format. Micrographs were collected on a 120 kv Tecnai Spirit microscope with a 4kx4k TemCam F416 camera using Leginon. Images were processed within Appion (26). Particles were selected with DoGpicker (27) and 2D classes were generated with MSA/MRA (28). Relion (29) was used for 3D classification followed by final 3D refinement with the particles most resembling HA/Fab complex.

### Peptide fragmentation and deuterium exchange mass spectrometry.

To maximize peptide probe coverage, the optimized quench condition was determined prior to deuteration studies. The HA head domain was diluted with buffer of 8.3 mM Tris, 150 mM NaCl, in H_2_O, pH 7.5) at 0°C and then quenched with 0.8% formic acid (v/v) containing various concentration of GuHCl (0.8–6.4 M) and Tris(2-carboxyethyl)phosphine (TCEP) (0.1 or 1.0 M). After incubating on ice for 5 minutes, the quenched samples were diluted 3-fold with 0.8% formic acid (v/v) containing 16.6% (v/v) glycerol and then were frozen at –80°C until they were transferred to the cryogenic autosampler. Using the quench buffer of 6.4 M GuHCl, 1 M TCEP in 0.8% formic acid gave an optimal peptide coverage map. The samples later were thawed automatically on ice and then immediately passed over an AL-20-pepsin column (16 μL bed volume, 30 mg/mL porcine pepsin [Sigma-Aldrich]). The resulting peptides were collected on a C18 trap and separated a C18 reversed phase column (0.2 × 50 mm, Optimize Technologies Inc.) running a linear gradient of 0.046% (v/v) trifluoroacetic acid, 6.4% (v/v) acetonitrile to 0.03% (v/v) trifluoroacetic acid, 38.4% (v/v) acetonitrile over 30 minutes with column effluent directed into an Orbitrap Elite mass spectrometer (Thermo Fisher Scientific). Data were acquired in both data-dependent MS:MS mode and MS1 profile mode. Proteome Discoverer software (Thermo Fisher Scientific) was used to identify the sequence of the peptide ions. HDEXaminer software (Sierra Analytics Inc.) was used for the analysis of the mass spectra. Fab-bound HAs were prepared by mixing H7-235 Fab with monomeric H7 head domain at a 1:1.1 (HA:Fab) stoichiometric ratio. The mixtures were incubated at 25°C for 30 minutes. All functionally deuterated samples, except for the equilibrium-deuterated control, and buffers were prechilled on ice and prepared in the cold room. Functional deuterium-hydrogen exchange reaction was initiated by diluting free HA or antibody-bound HA stock solution with D2O buffer (8.3 mM Tris, 150 mM NaCl, in D2O, pDREAD 7. 5) at a 1:2 vol/vol ratio. At 10 seconds, 100 seconds, or 1,000 seconds, the quench solution was added to the respective samples and incubated on ice for 5 minutes, then diluted 3-fold with 0.8 % formic acid before freezing at –80°C. In addition, nondeuterated samples and equilibrium-deuterated back-exchange control samples were prepared as previously described (30, 31). The centroids of the isotopic envelopes of nondeuterated, functionally deuterated, and fully deuterated peptides were measured using HDEXaminer and then converted to corresponding deuteration levels with corrections for back exchange (32).

### Statistics.

The descriptive statistics (mean ± SEM or mean ± SD) were determined for continuous variables as noted. Survival curves were estimated using the Kaplan Meier method and compared using the log rank (Mantel-Cox) test right censored, if they survived until the end of the study. Statistical analyses were performed using Prism v10.3.1 (GraphPad).

### Study approval.

For animal experiments, BALB/c mice were purchased from The Jackson Laboratories (Strain #000651). This study was conducted in the AAALAC-accredited laboratory animal research center of Vanderbilt University Medical Center. Breeding, maintenance, and experimentation complied with Vanderbilt Institutional Animal Care and Use Committee regulations (IACUC Protocol # M1900003-01). Details pertaining to mouse husbandry can be found in the “In vivo protection study” subsection.

### Data availability.

All data needed to evaluate the conclusions are present in the paper or the Supplemental Information. Values for all data points in graphs and figures are reported in the Supporting Data Values file. Further information and requests for resources and reagents should be directed to and will be fulfilled by the Lead Contact, James E. Crowe, Jr. (james.crowe@vumc.org). The crystal structure has been deposited in the Protein Data Bank with the accession number 9BT5.

## Author contributions

IMG, JD, and JEC designed the research studies. EA provided reagents. JD, IMG, HLT, SL, and RPI conducted experiments and acquired data. IMG, JD, CDB, ABW, RHC and JEC analyzed data. IMG and JEC wrote the manuscript. All authors reviewed, edited, and approved the final manuscript. JEC obtained funding.

## Supplementary Material

Supplemental data

Supporting data values

## Version 1 06/05/2025

In-Press Preview

## Version 2 07/08/2025

Electronic publication
