# Supplementary material for: Pan-H7 influenza human antibody virus neutralization depends on avidity and steric hindrance
Source: JCI Insight. 2025 Jun 5;10(13):e186182. doi: 10.1172/jci.insight.186182 (PMC12288907; doi:10.1172/jci.insight.186182)
Supplement: Supplemental data [file jciinsight-10-186182-s230.pdf]

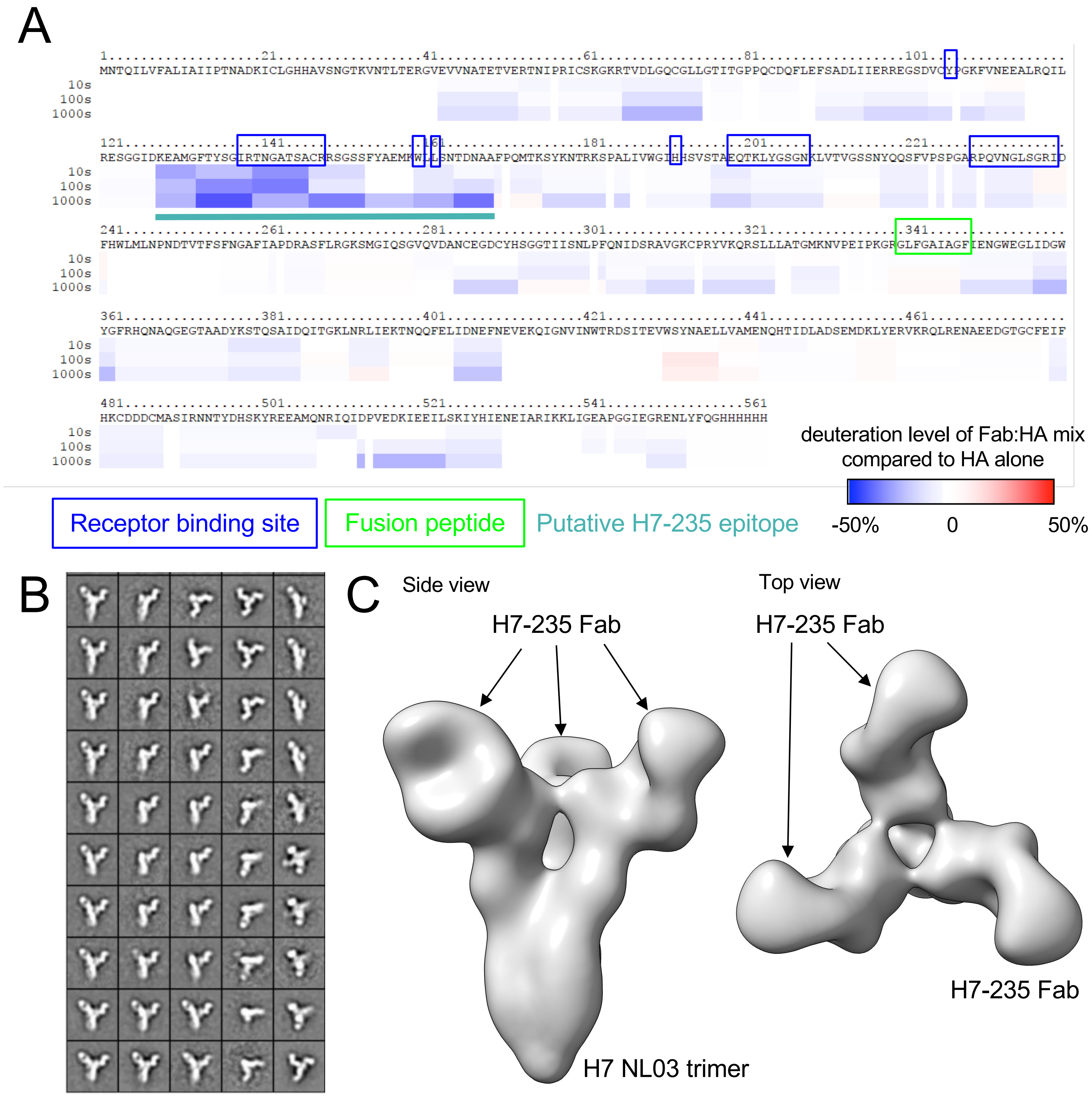

Figure S1. Hydrogen deuterium exchange mass spectrometry and negative staining electron microscopy data to determine the putative epitope on H7 HA binding by mAb H7-235. Related to Figure 1.

(A) Influence of mAb H7-235 on deuteration level of soluble trimer H7 HA (SH13 H7N9). The amino acid sequence of the H7 HA is shown with a ribbon diagram indicating differences in deuterium uptake. Blue colors indicating slower deuterium exchange in the presence of H7-235 Fab, while red colors indicate faster deuterium exchange in the presence of H7-235 Fab. Data are shown for 10 s, 100 s, or 1,000 s of deuterium labeling. HA residues are numbered from the start of the H7 HA construct.

(B) Representative subset of single particle nsEM 2D class averages shown with inverted contrast displays H7-235 Fab bound to TPCK-trypsin cleaved H7 A/Netherlands/219/2003 HA.

(C) Reconstructed 3D nsEM map of H7-235 with H7 HA soluble ectodomain trimer from A/Netherlands/219/2003 H7N7.

Heavy chain

```
                      FR1-IMGT                   CDR1-IMGT     FR2-IMGT        CDR2-IMGT                FR3-IMGT                              CDR3-IMGT         FR4-IMGT
                       (1-26)                     (27-38)       (39-55)         (56-65)                 (66-104)

              1        10        20          30           40        50          60           70        80        90        100                          120
              .........|.........|......     ...|........ .|.........|.....     ....|.....   ....|.........|.........|.........|....   ..            .....  ..|........
IGHV3-33*01   QVQLVESGG.GVVQPGRSLRLSCAAS     GFTF....SSYG MHWVRQAPGKGLEWVAV     IWYD..GSNK   YYADSVK.GRFTISRDNSKNTLYLQMNSLRAEDTAVYYC   AR            YGMDV  WGQGTLVTVSS
H7-235rev     QVQLVESGG.GVVQPGRSLRLSCAAS     GFTF....SSYG MHWVRQAPGKGLEWVAV     IWYD..GSNK   YYADSVK.GRFTISRDNSKNTLYLQMNSLRAEDTAVYYC   ARNGERWRVEDY YYGMDV  WGQGTLVTVSS
H7-235        QVQLVESGG.GVVQPGESLRLSCAAS     GFIF....RTYG MHWVRQAPGKGLEWVGV     IWYD..GSNK   YYGDSVK.GRFTISRDNSQNTLYLQMNSLRFEDTAVYYC   ARNGERWRVEDY YYGMDV  WGQGTLVTVSS
```

Light chain

```
                      FR1-IMGT                   CDR1-IMGT     FR2-IMGT          CDR2-IMGT               FR3-IMGT                       CDR3-IMGT   FR4-IMGT
                       (1-26)                     (27-38)       (39-55)           (56-65)                (66-104)

              1        10        20          30           40        50          60           70        80        90        100         110      120
              .........|.........|......     ...|........ .|.........|.....     ....|.....   ....|.........|.........|.........|....   .....|.    ..|.......
IGKV2-28*01   DIVMTQSPLSLPVTPGEPASISCRSS     QSLLHS.NGYNY LDWYLQKPGQSPQLLIY     LG.......S   NRASGVP.DRFSGSG..SGTDFTLKISRVEAEDVGVYYC   MQALQTP    FGQGTRLEIK IGKJ5*01
H7-235 rev    DIVMTQSPLSLPVTPGEPASISCRSS     QSLLHS.NGYNY LDWYLQKPGQSPQLLIY     LG.......S   NRASGVP.DRFSGSG..SGTDFTLKISRVEAEDVGVYYC   MQALQTPIT  FGQGTRLEIK
H7-235        EIVMTQSPLSLPVTPGEPASISCRSS     QSLLHS.NAHNY LDWYLQKPGQSPQLLIY     LG.......S   NRASGVP.DRFSGSG..SGTDFTLKISRVEAEDVG YYC   MQWLQTPIT  FGQGTRLEIK
```

Figure S2. Germline sequence analysis and germline revertant for H7-235. Related to Figure 3A. Alignment of IGHV3-33*01/IGKV2-28*01 germline sequences to the mAbs H7-235 and H7-235 revertant (H7-235rev). Mutations unique for H7-235 are highlighted in blue. The residues highlighted in red and orange represent the paratope in a heavy and light chains, respectively.

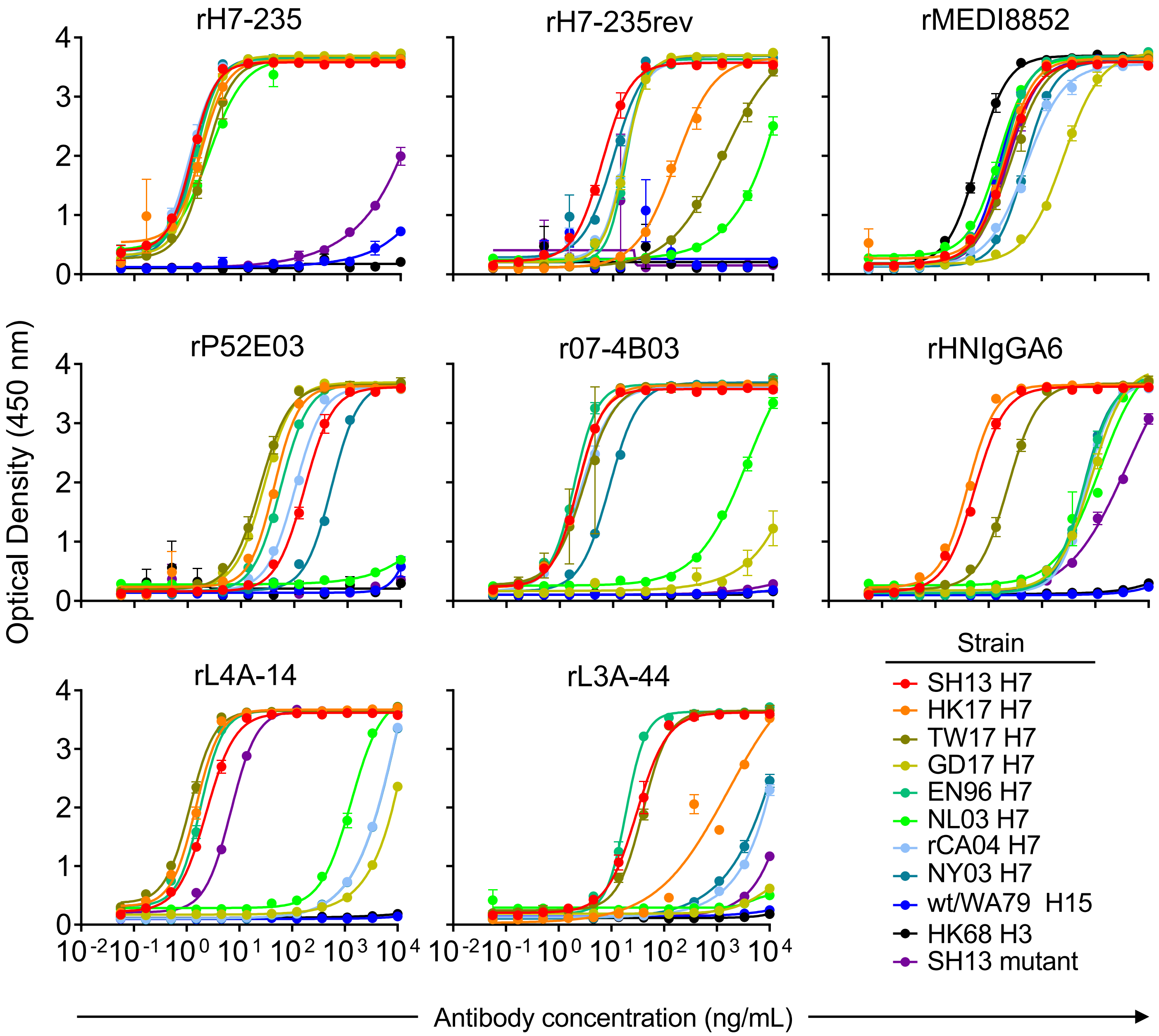

Figure S3. Concentration dependent curves used for effective concentration calculations in Figure 3A.

MAbs rH7-235 IgG1, rH7-235rev IgG1, rMEDI8852 IgG1, rP52E03 IgG1, r07-4B03 IgG1, rHNIgGA6 IgG1, rL4A-14 IgG1, rL3A-44 IgG1 were tested in three-fold serial dilutions for binding with recombinant soluble proteins from A/Shanghai/02/2013 (SH13 H7N9), A/Hong Kong/1/2017 (HK17 H7N9), A/Taiwan/01/2017 (TW17 H7N9), A/Guangdong/8H324/2017 (GD17 H7N9), A/England/268/1996 (EN96 H7N7), A/Netherlands/219/2003 (NL03 H7N7), A/Canada/rv504/2004 (rCA04 H7N3), A/New York/107/2003 (NY03 H7N2), A/shearwater/Western Australia/2576/1979 (wtWA79 H15N9), A/Hong Kong/1/1968 (HK68 H3N2), and triple mutant R141G/S145P/S146P on SH13 H7N9 background. Data represents one of two independent experiments, shown as mean ± SD of assay triplicates.

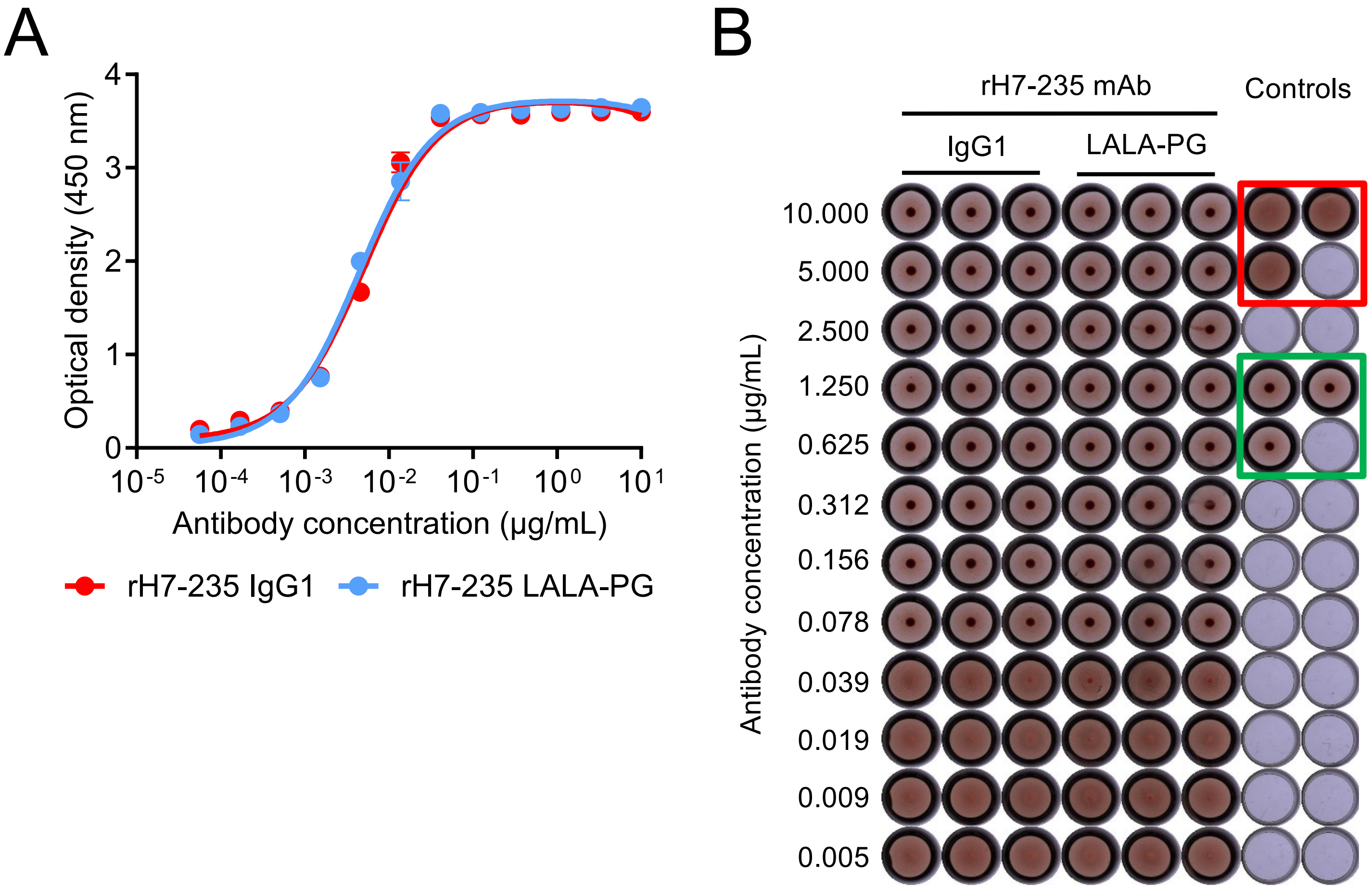

Figure S4. rH7-235 LALA-PG and rH7-235 IgG1 have comparable reactivity and HAI *in vitro*, related to Figure 4.

(A) MAbs rH7-235 IgG1 and rH7-235 LALA-PG were tested in three-fold serial dilutions for binding with recombinant soluble proteins from A/Shanghai/02/2013 (SH13 H7N9), shown as mean ± SD of assay triplicates.

(B) rH7-235 IgG1 and rH7-235 LALA-PG were tested in serial dilutions for hemagglutination inhibition of A/Shanghai/02/2013 H7N9; red boxes indicate virus and 1% turkey red blood cells without mAb, and green boxes indicate controls with only 1% turkey red blood cells.

SUPPLEMENTAL TABLE

Table S1. Data collection and refinement statistics for the crystals of H7-235/H7-HA1 complexes, Related to Figure 1

Data collection	
Crystal	H7-235/H7-HA1
PDB ID	9BT5
Wavelength (Å)	0.97872
Space group	C 1 2 1
Unit cell dimensions	
a, b, c (Å)	240.6, 95.05, 99.26
α, β, γ	90, 95.85, 90
Resolution (Å)	49.37 – 2.50
Unique reflections	76766 (11112)
Redundancy	3.9 (3.9)
Completeness (%)	99.6 (99.2)
R_{merge} (%)	9.6 (86.2)
I/σ(I)	9.9 (1.7)
Refinement statistics	
R_{factor} (%)	19.50
R_{free} (%)	25.10
R.m.s.d. (bond) (Å)	0.010
R.m.s.d. (angle) (deg)	1.113
Ramachandran plot	
Favored (%)	94.50
Allowed (%)	5.04
Outliers (%)	0.46

$R_{merge} = \Sigma \Sigma |I_{hkl} - I_{hkl(j)}|/\Sigma I_{hkl}$, where $I_{hkl(j)}$ is the observed intensity and I_{hkl} is the final average intensity.
$R_{work} = \Sigma ||Fobs| - |Fcalc|/\Sigma |Fobs|$ and $R_{free} = \Sigma ||Fobs| - |Fcalc||/\Sigma |Fobs|$, where R_{free} and R_{work} are calculated using a randomly selected test set of 5% of the data and all reflections excluding the 5% test set, respectively. Numbers in parentheses are for the highest resolution shell.
